# Transient dermatomyositis-like reaction following COVID-19 messenger RNA vaccination

**DOI:** 10.1016/j.jdcr.2023.05.028

**Published:** 2023-06-01

**Authors:** Rodrigo A. Gutierrez, Kari Connolly, Andrew Gross, Anna Haemel

**Affiliations:** a School of Medicine, University of California, San Francisco; b Department of Dermatology, University of California, San Francisco; c Department of Rheumatology, University of California, San Francisco

**Keywords:** autoimmune disease, dermatomyositis, drug side effects, mRNA vaccine, type 1 interferons, vaccine reaction

## Introduction

The spectrum of cutaneous immune responses previously observed with type I interferon therapy for melanoma and hepatitis C as well as the remarkable effectiveness of Janus Kinase inhibitors in dermatology highlight the central role of type I interferons in inflammatory skin disease.^1^^,^^2^ Early in the COVID-19 pandemic, reports of “pandemic chilblains” provided dermatologic indicators as to the importance of type I interferon signaling in the COVID-19 immune response; a patient with COVID-19 myopathy was subsequently found to have features of a type I interferonopathy, with pathogenesis akin to what is seen in idiopathic dermatomyositis (DM).^3^^,^^4^ Multiple case reports are now emerging of new or flaring DM associated with COVID-19 infection and/or messenger RNA (mRNA) vaccination.^5^ Although causality is difficult to establish, the key role of type I interferons in both COVID-19 and DM provides biologic plausibility for such an association. Notably, COVID-19-induced autoimmunity appears to be temporary in at least some patients; a transient inflammatory response may thus represent one clue toward a COVID-19 viral/vaccine-related trigger.^6^ Here, we report a classic, skin biopsy-supported DM-like reaction that occurred 2 weeks after COVID-19 mRNA vaccination and lasted 3 to 4 months before fully and spontaneously resolved.

## Case report

**Fig 1:**
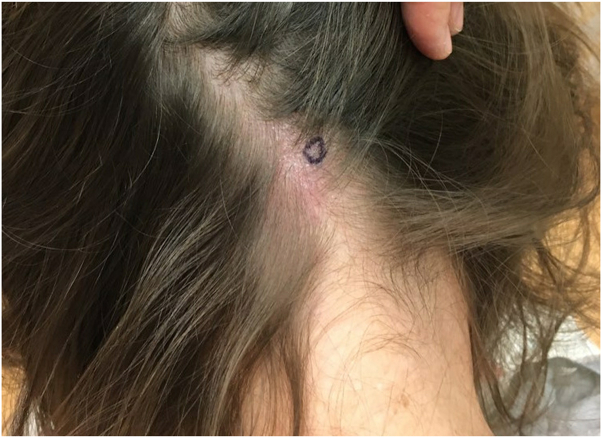
Image of scalp showing violaceous, psoriasiform erythema.

**Fig 2:**
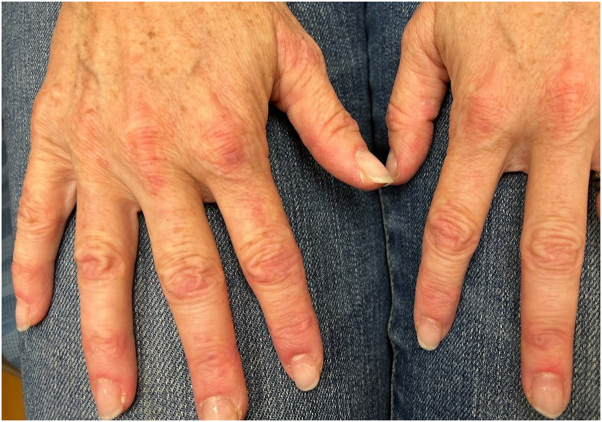
Characteristic Gottron’s papules over the dorsal aspects of the metacarpophalangeal and proximal interphalangeal joints.

**Fig 3:**
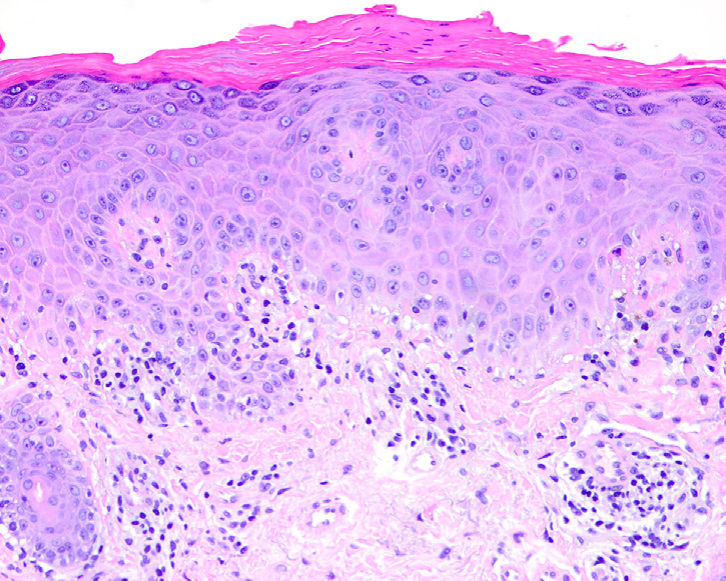
Hematoxylin-eosin stain demonstrating interface dermatitis with dermal mucin. Direct immunofluorescence (not shown) demonstrated granular deposits of immunoglobulin M along DEJ (100×). Photo courtesy of Dr. Rony A. Francois

**Fig 4:**
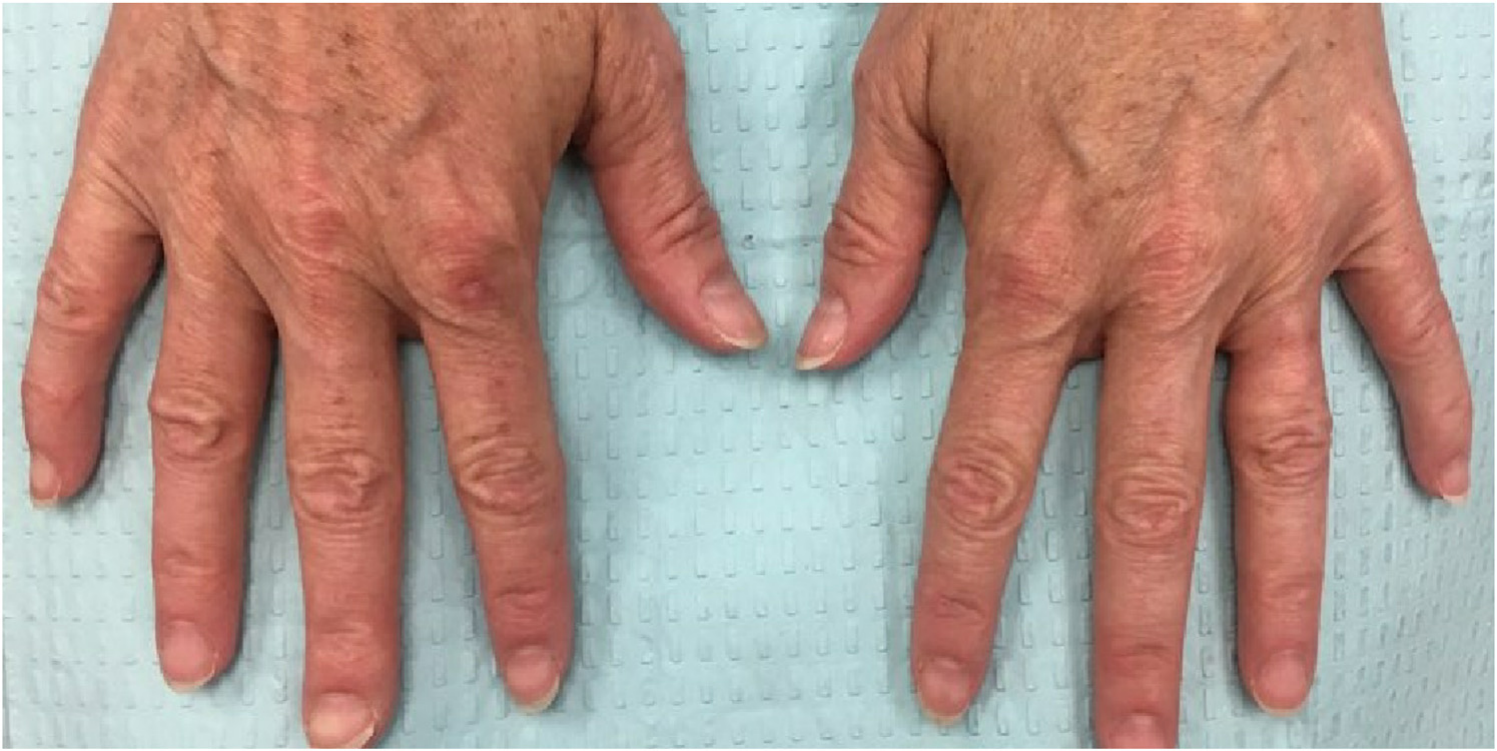
Resolution of Gottron’s papules at 4-month follow-up.

A 50-year-old woman with no known history of COVID-19 infection presented with myalgias, muscle weakness, and skin eruption beginning 2 weeks after her Pfizer/BioNTech BNT162b2 booster. She had previously received a single dose of the Johnson & Johnson vaccine without side effects. COVID-19 polymerase chain reaction (PCR) was negative at symptom onset. There was no associated weight loss, cough, or dysphagia. Medical history included a single pneumonia 6 months prior as well as mild ulcerative colitis managed with dietary measures; colonoscopy and all other routine screenings were up to date. The patient was on no medications at symptom onset. Examination revealed erythematous lichenoid papules over the digits and elbows and a bilateral violaceous eruption over the lateral aspect of the thighs, consistent with Gottron’s papules, Gottron’s sign, and Holster sign, respectively. Examination also revealed violaceous psoriasiform erythema on scalp, but heliotrope eruptions, cuticular changes, and gross muscle weakness were absent (Figs 1 and 2). Workup revealed a creatine kinase (CK) >2700, antinuclear antibody (ANA) 1:160 with homogenous staining, and a negative limited myositis antibody panel, including Mi-2. Shave biopsy from the scalp demonstrated interface dermatitis with dermal mucinosis; shave biopsy from the right side of the second metacarpophalangeal joint similarly demonstrated interface dermatitis (Fig 3). Direct immunofluorescence for both sites revealed granular junctional deposition of C3 with immunoglobulin M also present for the hand biopsy; IgA/IgG were negative for both the sites. Given the clinicopathologic features concerning for DM, the patient was treated with a prednisone taper from 40 mg per day to off over several weeks with brisk and full normalization of muscle enzymes. The skin eruptions, particularly over the thighs, were slower to resolve, lasting several months. At 4 months from initial presentation, the patient reported resolution of all symptoms, including her skin lesions off treatment with follow-up laboratory testings demonstrating normal CK and aldolase and negative myositis antibody panel, including Mi-2, MDA-5, TIF-1 gamma, and NXP-2 (Fig 4).

## Discussion

Multiple infectious triggers have been implicated in the onset of DM, such as parvovirus B19, hepatitis, and Epstein–Barr virus as well as toxoplasmosis; COVID-19/COVID-19 mRNA vaccinations, and other viral triggers, may produce a surge in type I interferons.^7^, ^8^, ^9^ Type I interferons, in turn, have the potential to promote or unmask autoimmunity in susceptible patients and/or in conditions where type I interferon signaling is central to disease pathogenesis, including DM.^1^ A real-world study in an autoimmune dermatology clinic further demonstrated that patients with existing DM were more likely to flare post COVID-19 vaccination than those with lupus.^5^ It is important to note that while such reactions to vaccines appear to be rare (at the level of case reports), earlier in the pandemic COVID-19-associated inflammatory myopathies, which share features with DM, may have affected up to one-third of COVID-19 patients.^4^^,^^10^

Regarding the relationship between COVID-19 and COVID-19 myopathy, a broad spectrum ranging from an acute worsening of existing myositis to chronic, postinfectious myalgic syndromes have been reported in infected patients, with anti-MDA5 antibody titers trending with severity.^4^^,^^11^ In previously reported cases of myositis induced by COVID-19 and/or COVID-19 vaccine, interface dermatitis has been a prominent feature.^12^ Prior cases of COVID-19 vaccine-associated DM involved an onset 5 to 6 days after vaccination, considerably earlier than peak antibody response induced by the vaccine, and a longer recovery period with more aggressive treatment compared with this case.^13^^,^^14^

The timing and self-resolving nature of the DM-like reaction in the current patient may suggest transient COVID-19/COVID-19 vaccine-related autoimmunity. First, the patient’s symptoms occurred roughly 2 weeks after administration of her booster, which aligns temporally with the peak antibody response of mRNA vaccines in healthy patients.^15^ Second, the patient’s symptoms resolved within a short time course and with minimal treatment. Typically, to be considered a drug side-effect: (1) symptoms must be temporally related, (2) withdrawal should lead to clearance, and (3) rechallenge should induce recurrence; this case meets conditions 1 and 2 above, and any association could likely be deemed possible or probable but not certain.

In summary, we present a transient DM-like reaction following COVID-19 mRNA vaccination, which may have been triggered by a type I interferon surge and then progressively resolved over several months with minimal treatment. In retrospect, the presence of immunoglobulin M deposits alone on the direct immunofluorescence skin biopsy may have provided some indication of the acuity of this process. However, the passage of time has been the most reliable indicator that this reaction has (and remains) resolved.

## Conflicts of interest

Dr Haemel reports serving as a consultant to CSL Behring and Guidepoint. Authors Gutierrez, Connolly, and Gross have no conflicts of interest to declare.
